# Stage-specific digital health technology biomarkers enhance diagnostic and early progression detection in Parkinson’s disease

**DOI:** 10.3389/fneur.2026.1869945

**Published:** 2026-07-08

**Authors:** Matthew D. Czech, Samantha Sawicki, Cindy Zadikoff, Chengcheng Liu, Weining Robieson, Ying Liu, Weihua Shi, Jie Shen, Michelle Crouthamel, Maria S. Quinton, Josh Cosman, E. Ray Dorsey, Jamie L. Adams, Naomi Nevler

**Affiliations:** 1 AbbVie, North Chicago, IL, United States; 2 Department of Neurology, University of Rochester Medical Center, Rochester, NY, United States

**Keywords:** composite score, diagnosis, digital health, objective biomarker, progression, wearables

## Abstract

**Background:**

Digital health technology measurements show promise as objective biomarkers in Parkinson’s disease. However, their sensitivity and consistency across early disease stages remain poorly defined, which currently limits their utility in clinical trial design.

**Objectives:**

The study aims to evaluate the effectiveness of various DHT measures within tremor, bradykinesia, and axial symptom domains, in differentiating early stage PD from healthy controls and in monitoring short-term disease progression.

**Methods:**

In this study, we examined a range of tremor, bradykinesia, and axial symptom measures across age-matched healthy volunteers (*n* = 45), newly diagnosed Parkinson’s disease patients (*n* = 54), and participants after 12 months of follow-up (*n* = 40) from WATCH-PD, a multicenter, observational study.

**Results:**

Our findings reveal distinct categories of functional measures: some effectively differentiate healthy controls from patients with recent Parkinson’s diagnosis but show limited sensitivity to early progression, while others are insensitive to initial diagnosis yet capture longitudinal change. Models trained on disease-stage specific feature sets were most effective for their intended task, with the performance gain over combined features being more pronounced for progression detection (∆AUC = 0.15, ∆Cohen’s *d* = 0.72).

**Conclusion:**

These findings underscore the need to align composite digital biomarker design and feature selection to both disease stage and clinical objective, and suggest that adaptive, symptom- and side specific DHT measures may enhance sensitivity in trial population selection and short-term progression monitoring.

## 1 Introduction

Despite decades of research, there are currently no approved disease modifying treatments in early Parkinson’s disease (ePD) (1). Designing such trials is challenging, in part, due to the slow rate of change on conventional clinical scales, the absence of reliable fluid or imaging biomarkers for disease progression or target engagement, and the confounding effects of symptomatic therapies (2, 3). Accurate functional clinical diagnosis and progression monitoring is therefore essential, yet it is hindered by the fact that measurable functional symptoms typically emerge only after significant neuronal loss and are subject to considerable patient heterogeneity, complicating study design (4, 5). Furthermore, conventional scales like the Movement Disorder Society Unified Parkinson’s Disease Rating Scale (MDS-UPDRS) are subjective, point-in-time (episodic) measures, and insensitive to early changes, contributing to lengthy and expensive trials prone to failure (6, 7).

Improving diagnostic tools and progression monitoring in ePD is a recognized priority. The absence of validated biomarkers and the limitations of current clinical scales underscore the need for objective, sensitive tools that can quantify disease status with high resolution (8, 9). Recognizing these challenges, recent efforts have explored wearable sensor based digital health technologies (DHTs) to complement or improve on traditional scales (10, 11). Large observational cohorts have demonstrated the potential of inertial sensor data from smartphones, smartwatches, and dedicated wearables to capture fine motor, gait, tremor, and speech metrics (12, 13). These digital measures can detect progression over 12 months and often exhibit greater sensitivity than conventional instruments (14–19).

Despite these advances, many digital studies are still restricted by the limited set of features available on their data acquisition platforms and by assumptions of linear change across disease progression. In particular, the role of domain specific (tremor, axial, bradykinesia) and side specific (more affected vs. less affected limb) measures across disease stages has been underexplored (20). The present study addresses these limitations by applying advanced algorithms to derive functional measures from key performance tasks in axial, tremor, and bradykinesia domains and examine the linearity of measure progression across age matched healthy volunteers (HV), ePD participants at baseline, and the same cohort after 12 months across domains and sides using data from the multicenter, observational WATCH-PD (Wearable Assessment in The Clinic and at Home in PD) study. Importantly, we evaluate whether developing composite scores specific to disease stages improves sensitivity for both diagnosis and progression monitoring in early Parkinson’s disease.

## 2 Materials and methods

### 2.1 Participants and procedure

The multicenter, observational WATCH-PD study (Wearable Assessment in the Clinic and at Home in PD; NCT03681015) enrolled 82 individuals with early, untreated Parkinson’s disease (PD) and 50 age-matched controls aged over 30 years. Participants were recruited through clinics, research registries, and social media, and enrolled across 17 Parkinson Study Group sites. While the cohort was predominantly white and well educated—a limitation—its demographics aligned with similar large observational PD studies (21). Inclusion criteria for PD participants were: age ≥ 30 years, disease duration < 2 years, and Hoehn & Yahr stage ≤ 2. Major exclusions included prior dopaminergic or other PD medications and alternative parkinsonian diagnoses. The study was powered to detect a mean change of 6.9 in MDS-UPDRS Part III scores over 12 months, aiming for 30 participants completing follow-up without medication, accounting for up to 50% initiating dopaminergic therapy and a 15% dropout rate.

In-clinic assessments occurred at baseline and months 1, 3, 6, 9, and 12. Each participant wore five APDM Opal sensors (Clario, Inc.)—placed on both feet, both wrists, and the lower back—collecting triaxial accelerometer, gyroscope, and magnetometer data at 128 Hz. Investigators administered and scored the full MDS-UPDRS concurrently while patients were wearing the sensors. Validated algorithms were implemented to derive measures in axial (22, 23), tremor (24), and bradykinesia (10, 25) domains. Axial domain analysis focused on measures related to lower limb, lumbar, trunk, and upper limb movements during gait, as well as turning, derived from a 2-min casual walking assessment. Tremor analysis focused on upper limb tremor derived from continuous wrist sensor data collection across MDS-UPDRS resting, postural, and kinetic tremor assessments. Bradykinesia analyses focused on MDS-UPDRS pronation-supination and toe tapping tasks selected for robust signal capture via the wrist and foot sensors.

Following exclusion of participants that initiated symptomatic therapy during the study, sessions with missing clinical data, technical failures, or insufficient movement detection, the Baseline dataset used to evaluate feature relationships with MDS-UPDRS, between cohorts, and between visits included 54 PD (mean age 64.3 ± 9.3 years; 23 female; BMI 27.2 ± 4.7) and 45 HV participants (mean age 61.5 ± 9.8 years; 28 female; BMI 28.3 ± 6.8). At month 12, data were available from 40 PD patients. For modeling, we only included participants with all feature values available across all three domains as well as available data for both Baseline and 12-month visits, which resulted in a dataset consisting of 35 PD and 27 HV participants. A detailed flow diagram detailing exclusions at each step is provided in Supplementary Figure S1.

### 2.2 Ethics

The WCG™ Institutional Review Board approved (IRB Tracking #: 20183288) the procedures used in the study, and there was full compliance with human experimentation guidelines. All participants provided written informed consent before study participation.

### 2.3 Statistics and reproducibility

Cohen’s d coefficients and Wilcoxon *p* values (rank-sum test for between-group comparisons; signed-rank test with paired Cohen’s d for within-subject baseline to 12 month comparisons) were used to assess relationships between DHT measures across cohorts and timepoints. Measures in each domain (bradykinesia, tremor, axial) were compared using Spearman correlation with their relevant MDS-UPDRS sub-scores, where MDS-UPDRS bradykinesia sub-score was calculated by summing items 3.4–3.8 and 3.14, MDS-UPDRS tremor sub-score was calculated by summing items 3.15–3.18, and MDS-UPDRS axial sub-score was calculated by summing items 3.9–3.13.

We classified features into diagnostic, late progression, and continuously progressing categories using Cohen’s *d* thresholds of ≥ 0.25. Diagnostic features additionally required a progression effect size between −0.1 and 0.1, and conversely, progression features required a diagnostic effect size in that range to allow a small tolerance for change. For modeling, diagnostic and progression feature sets were selected based solely on the ≥ 0.25 Cohen’s *d* criterion, without the ±0.1 cross stage restriction. Features were included only if their direction of change was consistent with the expected correlation to the relevant MDS-UPDRS sub score, allowing a tolerance of ±0.1. The 0.25 Cohen’s d threshold was chosen as a slightly more conservative threshold than the 0.20 default guideline for” small” effect size (26).

Composite digital scores were derived via unweighted *z*-score summation. Each feature’s *z*-score was normalized (mean = 0, SD = 1) to the baseline HV value, directionally corrected, and summed to produce progression, diagnostic, and combined composite scores.

To estimate generalization performance while limiting overfitting from concurrent feature selection and model fitting on the same data, we used a nested 10-fold (outer) by 10-fold (inner) cross-validation procedure. Within each outer fold, Cohen’s d feature selection (top 10 features per category—diagnostic or progression—based on |Cohen’s d| ranking) and z-standardization (using healthy-volunteer training-fold mean and standard deviation) were re-derived using only training-fold subjects. For combined models, a variance filter was applied to the same training-fold subjects to retain the 10 highest-variance features. Feature sets and composite scores were evaluated with general linear model (GLM) logistic regression with Lasso (L1) regularization, with the regularization penalty tuned within the inner cross-validation loop. AUC values with 95% DeLong confidence intervals and Cohen’s d on prediction probabilities were computed on pooled out-of-fold predictions. Model comparisons employed DeLong’s test, applied as one-tailed for pre-specified directional hypotheses (diagnostic features > progression features for HV-vs-ePD-BL discrimination; progression features > diagnostic features for ePD-BL-vs-12 m discrimination). Per-feature univariate effect sizes (paired Cohen’s d for within-subject baseline-to-12-month comparisons; unpaired Cohen’s d for HV-vs-ePD comparisons) were tested with Wilcoxon signed-rank or rank-sum tests, with *p*-values corrected for multiple comparisons using the Benjamini–Hochberg false discovery rate (reported as q below).

## 3 Results

### 3.1 Distinct non-linear trajectories of functional measures across diagnosis and early progression in ePD

**Figure 1:**
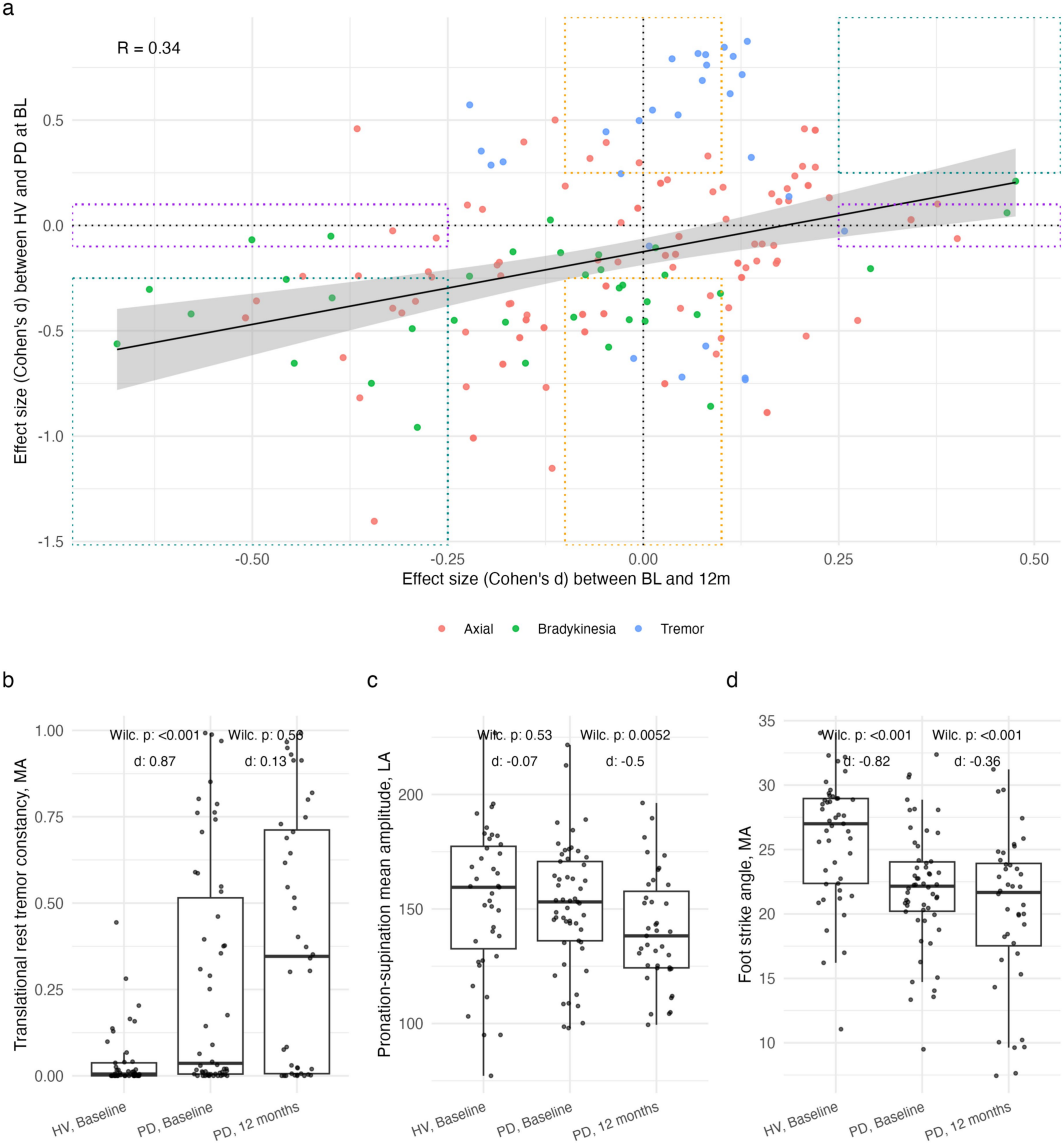
Distinguishing diagnostic, late progressing, and continuously progressing measures. **(a)** A subset of measures (dark cyan box) across axial, bradykinesia, and tremor domains demonstrate change between both HV and ePD participants at baseline (effect size > 0.25), and between ePD at baseline and 12 months (effect size > 0.25). Another subset (purple box) demonstrates change in ePD patients between baseline and 12 months (effect size > 0.25), but does not distinguish between HV and ePD patients (effect size < 0.1). A third subset (orange box) distinguishes HV and ePD patients (effect size > 0.25), but does not change in ePD patients across 12 months (effect size < 0.1). Examples of **(b)** diagnostic (more affected side translational rest tremor constancy), **(c)** late progressing (less affected side pronation-supination mean amplitude), and **(d)** continuously progressing (more affected side foot strike angle) measures are shown.

We examined functional measures across axial, tremor, and bradykinesia domains in their ability to distinguish between age-matched HV from ePD patients at baseline, and to track change from baseline to 12 months in ePD patients who did not start symptomatic medications for the duration of the study. Effect sizes between HV vs. ePD at baseline and ePD baseline vs. 12 months showed a moderate correlation (*r* = 0.34), suggesting that many features progress non-linearly across diagnosis and the first year of disease (Figure 1a). Measures clustered into three broad categories: diagnostic (diagnostic effect size > 0.25; progression effect size < 0.1), late progressing (diagnostic effect size < 0.1; progression effect size > 0.25), and continuously progressing (diagnostic effect size > 0.25; progression effect size > 0.25) within the context of ePD. Representative examples for each category are shown in Figures 1b–d.

Bradykinesia measures showed a clear task-dependent pattern: toe-tapping (TT) features served primarily as diagnostic markers, while pronation–supination (PS) features captured both baseline differences and ongoing within-subject decline. Among 10 strict-diagnostic bradykinesia features (5 MA, 5 LA), six were TT-derived (capturing amplitude, slope of amplitude, amplitude variability, mean velocity, and slope of velocity; strongest effect: MA TT amplitude variability, q = 0.009) and four were PS-derived (all frequency-related—mean and variability of frequency on both sides). Continuous progression in bradykinesia (*n* = 9) was dominated by PS features (8 of 9) and the MA side (6 of 9), led by MA-side slope features capturing accelerating task-related slowing (slope of MA pro-sup maximum velocity, paired d = −0.67, q = 0.050; slope of MA pro-sup amplitude, paired d = −0.63). The three strict-late-progression bradykinesia features were exclusively LA-derived—LA PS mean amplitude (paired d = −0.50, q = 0.082), LA PS maximum-velocity coefficient of variation (paired d = +0.47, q = 0.050), and LA TT slope of frequency (paired d = −0.40)—suggesting that the less-affected side begins to show task-related slowing only after diagnosis. The remaining 14 bradykinesia features fell outside these three strict categories because they showed moderate but sub-threshold effects (|Cohen’s d| typically in the 0.10–0.25 range) in at least one of the two dimensions, but they consistently aligned with the dominant pattern: TT amplitude features tended toward diagnostic behavior (e.g., MA TT mean amplitude HV-vs-ePD d = −0.65, BL-vs-12 m d = −0.15, just missing the strict diagnostic-only cutoff), while PS variability features tended toward continuous behavior (e.g., MA PS max-velocity coefficient of variation BL-vs-12 m d = +0.48, just below the strict continuous-progression cutoff for baseline effect). Together, these patterns position MA-side PS slope features as the strongest candidates for short-term progression monitoring, while TT amplitude features are most useful for case–control discrimination at baseline.

Tremor was a robust baseline signature in ePD but did not capture meaningful within-subject longitudinal change in this 12-month window. Of 28 tremor features examined, 12 met the strict diagnostic criterion (large baseline difference with negligible 12-month change), 0 met the continuous-progression criterion, and only 1 met the strict late-progression criterion. Of the 12 strict-diagnostic features, 7 were MA-side and 5 LA-side, with the most robust effects after FDR correction (q ≤ 0.04) on MA translational rest tremor amplitude (median and 95th-percentile log values), MA rest tremor frequency, MA rotational rest tremor amplitude (median and 95th-percentile log), and MA rotational rest tremor amplitude variability; LA-side diagnostic features captured similar translational rest tremor amplitude and frequency increases alongside active tremor amplitude differences. The single late-progression measure was LA rotational rest tremor constancy (paired d = +0.26, q = 0.70—not surviving multiple-comparison correction), which we hypothesize may reflect a gradual “catch-up” of the less-impaired side toward MA severity. The remaining 15 tremor features demonstrated moderate but sub-threshold effects (|Cohen’s d| typically 0.10–0.25 in at least one dimension) that did not meet the strict criteria for any of the three primary categories—but they extend the diagnostic-only pattern: most have strong baseline effects (|HV-vs-ePD d| up to 0.87—e.g., MA translational rest tremor constancy d = +0.87, MA rotational rest tremor constancy d = +0.85, MA translational rest tremor amplitude d = +0.80) paired with small but non-zero 12-month change (|BL-vs-12 m d| typically 0.11–0.22). Across categories, no tremor feature reached |paired d| > 0.25 between visits in either direction. Despite tremor’s prominence at presentation, inertial-sensor-derived tremor features in this cohort and window functioned as strong baseline markers rather than progression markers.

**Figure 2:**
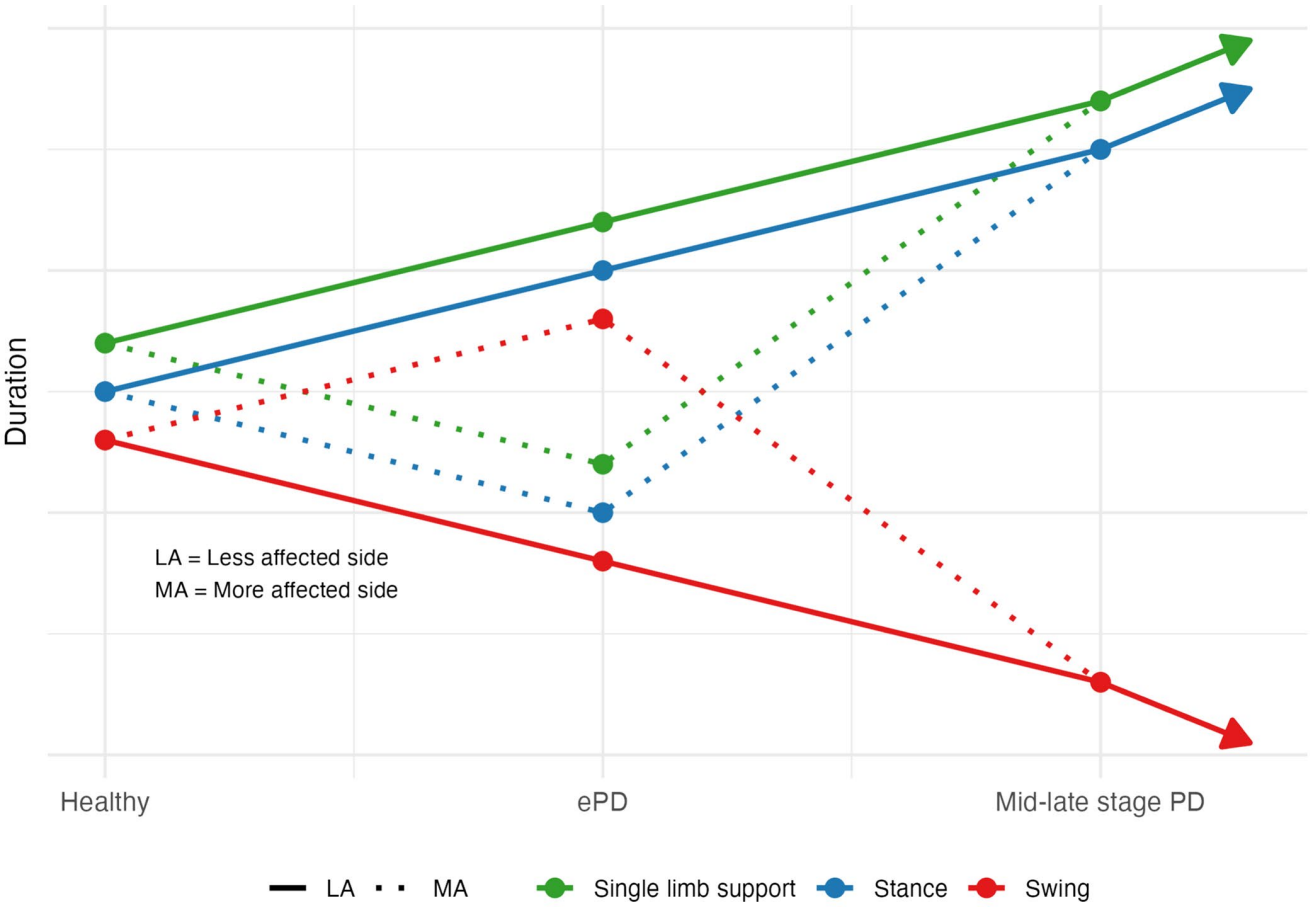
Projected gait measure trajectories, reflecting compensatory” limping” mechanics stemming from asymmetric motor impairment at onset.

In the axial domain, baseline features distinguishing HV from ePD spanned both gait and turn assessments, though only gait-derived features met the strict diagnostic-only criterion. Of 110 axial features examined, 18 met the strict diagnostic criterion (all gait-derived; 10 MA, 8 LA, plus side-independent trunk/lumbar measures), with the strongest effects after FDR correction on bilateral trunk sagittal range-of-motion variability (q = 0.005), MA arm swing velocity variability (q = 0.009), and bilateral coronal trunk and lumbar range-of-motion features (q = 0.043–0.046). A further 78 axial features showed moderate but sub-threshold effects in at least one dimension (|Cohen’s d| typically 0.10–0.25), several with very large baseline effects placed outside the strict diagnostic-only category because of small but non-zero 12-month change—most notably bilateral turn velocity mean (HV-vs-ePD d = −1.01, paired BL-vs-12 m d = −0.22), bilateral turn angle mean (d = −0.66 / −0.18), MA arm range-of-motion variability (d = −1.15 / −0.12), MA arm swing velocity mean (d = −0.77 / −0.12), and bilateral trunk coronal range-of-motion variability (d = −0.89 / +0.16). Strikingly, while PD is generally characterized by increased single limb support and stance duration with reduced swing time prompted by shuffling gait, MA and LA gait cycles in our cohort showed mirror-image asymmetries (Figure 2): MA swing duration was increased (Cohen’s d = +0.39, q = 0.076) while MA stance and single-limb-support durations were reduced (d = −0.39, q = 0.076; d = −0.19), with LA-side stance (d = +0.17) and single-limb-support (d = +0.32, q = 0.149) showing the opposite pattern. Additional MA-vs-LA asymmetries included increased MA step duration (d = +0.46, q = 0.011) and reduced MA foot strike angle (d = −0.82, q = 0.001) and arm swing range of motion (d = −1.40, q < 0.001), consistent with longer, flatter steps and minimal arm swing on the MA side reflecting compensatory limping in response to unilateral motor impairment at onset. These cycle-time inversion features themselves showed small, sub-threshold within-subject changes over 12 months (paired |d| < 0.20 for all timing means), but trended in directions consistent with progression toward the bilateral shuffling gait pattern commonly observed in later-stage PD (37)—both sides showed decreased swing duration (LA paired d = −0.18, MA d = −0.05) and increased stance duration (LA d = +0.18, MA d = +0.05), with the larger LA-side magnitudes consistent with a gradual catch-up of the less-affected side as the disease progresses.

Axial progression in the first year tracked two distinct feature classes: gait-cycle variability emerged predominantly on the less-affected side as a late-progression signature, while bilateral mean amplitude features—foot strike angle and arm swing range of motion—showed continuous progression on both sides. Five strict-late-progression measures (4 LA, 1 MA) uniformly captured gait-cycle variability (LA foot strike angle, swing- and stance-time variability, toe-out angle variability; MA circumduction variability; paired |d| = 0.26–0.40), indicating that increased gait variability is a longitudinal signal not present at baseline. Nine continuous-progression measures demonstrated both baseline differences and 12-month deterioration, led by MA arm swing range of motion (q < 0.001 for HV-vs-ePD; paired d = −0.34, q = 0.005 for BL-vs-12 m), MA foot strike angle (q = 0.001 / q = 0.039), bilateral turn-velocity variability (paired d = −0.51, q = 0.050 each), MA lateral step variability (paired d = −0.49, q = 0.050), bilateral gait speed mean, and LA foot strike angle mean. Several axial features showed continuous-leaning patterns just below the strict 12-month threshold—most notably bilateral turn velocity mean (HV-vs-ePD d = −1.01, paired BL-vs-12 m d = −0.22) and LA arm swing velocity variability (d = −0.77/−0.23)—suggesting additional candidate progression markers in this borderline range. Together with the gait-inversion findings above, these results indicate a transition from baseline asymmetry—captured by mean cycle-time inversions—to bilateral and variability-based axial deterioration during the first year, with foot strike angle, arm swing range of motion, and LA gait variability as the principal candidate markers for monitoring axial progression in this window.

### 3.2 Functional measures show differential utility as diagnostic versus progression markers in ePD

To examine how functional measures vary in sensitivity across disease stages, we developed predictive GLM logistic regression models to (1) distinguish HV from early Parkinson’s disease (ePD) at baseline, and (2) differentiate ePD at baseline from 12-month follow-up. Diagnostic models were built using the top 10 measures most sensitive to disease status at baseline (regardless of progression sensitivity), both as a Lasso-regularized logistic regression and as a summed z-score composite. Progression models were similarly built from the top 10 measures distinguishing ePD baseline from 12 months (regardless of diagnostic sensitivity). Combined models took the union of the progression and diagnostic top-10 lists and retained the 10 highest-variance features (variance computed on z-standardized values in each training fold). Generalization performance was estimated using nested 10-fold (outer) by 10-fold (inner) cross-validation, with feature selection and z-standardization re-derived within each training fold; AUCs with 95% DeLong confidence intervals are reported on pooled out-of-fold predictions, and within-cohort apparent estimates are noted alongside for reference.

**Figure 3:**
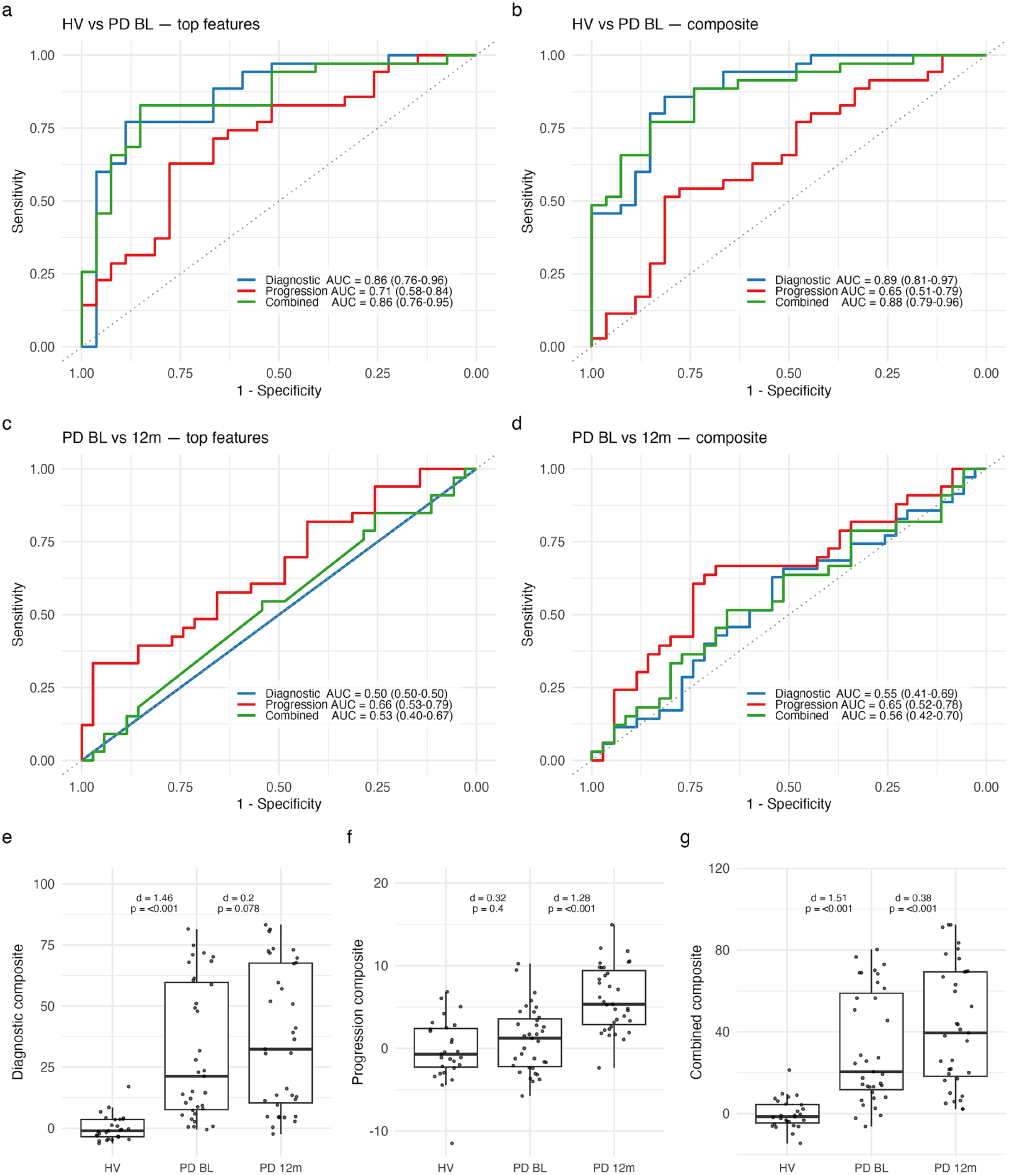
Functional measurement models optimized for diagnosis or progression are most effective when used for their respective purposes. **(a,b)** Comparison of GLM logistic regression models built using top features or composite score for distinguishing HV and PD at Baseline. **(c,d)** Comparison of GLM logistic regression models built using top features or composite score for distinguishing PD at Baseline from 12 month follow-up. **(e–g)** Boxplots demonstrating raw composite score distributions across cohorts and timepoints, including Cohen’s d `and Wilcoxon *p* values.

For HV-vs-ePD-BL discrimination, diagnostic feature sets achieved the strongest performance: top diagnostic features yielded an AUC of 0.86 (95% CI 0.76–0.96; apparent AUC = 0.97) and the diagnostic composite score 0.89 (95% CI 0.81–0.97; apparent AUC = 0.92) (Figures 3a,b). These significantly outperformed progression-based models (top progression features: AUC = 0.71, 95% CI 0.58–0.84; progression composite: 0.65, 95% CI 0.51–0.79; one-tailed DeLong *p* = 0.030 and 0.003, respectively). Diagnostic models performed comparably to combined-feature models (combined top features: AUC = 0.86, 95% CI 0.76–0.95; combined composite: 0.88, 95% CI 0.79–0.96; both DeLong *p* > 0.25 vs. diagnostic)—likely driven by inclusion of high-sensitivity diagnostic measures such as arm swing range of motion during gait (Table 1) in both the diagnostic and combined feature sets.

**Table 1:** Diagnostic measures, ordered by effect size between HV and ePD at baseline.

Domain	Measure	Side	ePD BL vs. 12 m	HV vs. ePD
Axial	Arm swing ROM	More affected	−0.29	−1.40
Axial	Arm swing ROM variability	More affected	−0.14	−1.15
Bradykinesia (pro-sup)	Maximum rotational velocity	More affected	−0.16	−0.96
Tremor	Translational rest tremor constancy	More affected	0.35	0.87
Tremor	Rotational rest tremor constancy	More affected	0.32	0.85
Axial	Foot strike angle	More affected	−0.33	−0.82
Tremor	Translational rest tremor amplitude (95th percentile)	More affected	0.25	0.82
Tremor	Translational rest tremor frequency	More affected	0.13	0.81
Tremor	Translational rest tremor amplitude (median)	More affected	0.27	0.80
Tremor	Rotational rest tremor amplitude (95th percentile)	More affected	0.23	0.79

For ePD-BL-vs-12 m discrimination, progression feature sets achieved the strongest performance: top progression features yielded an AUC of 0.66 (95% CI 0.53–0.79; apparent AUC = 0.83) and the progression composite score 0.65 (95% CI 0.52–0.78; apparent AUC = 0.81), outperforming diagnostic-based models (top diagnostic features: AUC = 0.50; diagnostic composite: 0.55, 95% CI 0.41–0.69) and combined-feature models (combined top features: 0.53, 95% CI 0.40–0.67; combined composite: 0.56, 95% CI 0.42–0.70) (Figures 3c,d). Under one-tailed DeLong’s tests, top progression features significantly outperformed top diagnostic features (*p* = 0.009) and combined top features (*p* = 0.049), while composite-score comparisons trended in the same direction but did not reach significance (*p* = 0.15 vs. diagnostic composite, *p* = 0.13 vs. combined composite).

Composite score distributions reflected the same pattern: diagnostic composites showed large baseline group separation (HV vs. ePD BL: d = 1.46, *p* < 0.001) but minimal progression sensitivity (BL vs. 12 m: paired d = 0.20, *p* = 0.08); progression composites showed the reverse trend (HV vs. ePD BL: d = 0.32, *p* = 0.40; BL vs. 12 m: paired d = 1.28, p < 0.001); combined composites paralleled the diagnostic composite at baseline (HV vs. ePD BL: d = 1.51, p < 0.001) but did not match the progression composite’s longitudinal sensitivity (BL vs. 12 m: paired d = 0.38, *p* < 0.001) (Figures 3e,g).

Overall, these findings show that functional measures optimized for diagnosis or progression are most effective when used for their respective purposes. For progression detection, top stage-specific features outperformed combined-feature models (DeLong p = 0.049 for top features); for diagnosis, stage-specific and combined feature sets performed comparably (DeLong p > 0.25), reflecting that combined models retain the same high-sensitivity diagnostic features (Table 1). This underscores that the optimal features for clinical outcome modeling may depend on both disease stage and the intended application.

A practical implication of these results is that unweighted composite scores yielded equivalent discrimination to weighted Lasso-regularized models under nested cross-validation (diagnostic: top features AUC = 0.86 vs. composite 0.89; progression: 0.66 vs. 0.65; combined: 0.86 vs. 0.88). For the construction of digital biomarker scores in early PD, this suggests that careful feature selection—identifying the right measures with strong diagnostic or progression signal—matters more than the specific weighting scheme used to combine them. Simple unweighted composites of well-selected features may therefore be preferable for deployment given their equivalent discrimination and greater interpretability, with optimization of weighting schemes deferred to studies with substantially larger samples.

The measures most strongly distinguishing HV from ePD had effect sizes ranging from 0.81 to 1.40 and were predominantly recorded on the MA side (9 of 10 features; Table 1). Of the 10 most sensitive diagnostic measures, five related to axial symptoms, four to tremor, and one to bradykinesia. MA side arm swing ROM (Cohen’s d = −1.40) and arm swing ROM variability (d = −1.15) showed particularly large differences between HV and ePD at baseline. In contrast, these measures exhibited only weak within-subject change between baseline and 12 months under paired testing (paired d = −0.34 and −0.12, respectively).

The measures showing the greatest progression from ePD baseline to 12 months had effect sizes ranging from 0.46 to 0.67 and were predominantly associated with bradykinesia and axial symptoms (Table 2). Of the 10 most sensitive progression measures, seven were linked to bradykinesia, three to axial symptoms, and none to tremor, with a 6:4 split between more and less affected sides. Negative Cohen’s d values for bradykinesia features in Table 2 indicate decreases in the corresponding measure over 12 months (e.g., reductions in pronation–supination amplitude, velocity, or frequency), consistent with the progressive slowing of early Parkinson’s disease, while positive values indicate increases (e.g., increased gait or step variability). The most responsive measures captured slope changes in amplitude, velocity, and frequency during the pronation–supination task—reflecting a slowing of movement with repetitions over the task duration as a key marker of disease progression. These measures demonstrated moderate effect sizes (paired d = 0.46–0.67) for detecting progression in ePD across 12 months, but generally weak to moderate effect sizes (|HV-vs-ePD d| = 0.06–0.56) for distinguishing HV from ePD at baseline.

**Table 2:** ePD progression measures, ordered by effect size between ePD at Baseline and 12 month follow up.

Domain	Measure	Side	ePD BL vs. 12 m	HV vs. ePD
Bradykinesia (pro-sup)	Slope change in rotational amplitude	More affected	−0.79	−0.30
Bradykinesia (pro-sup)	Slope change in rotational velocity	More affected	−0.65	−0.56
Bradykinesia (pro-sup)	Slope change in frequency	Less affected	−0.56	−0.42
Bradykinesia (pro-sup)	Slope change in rotational velocity	Less affected	−0.52	−0.34
Bradykinesia (pro-sup)	Rotational amplitude (mean)	Less affected	−0.42	−0.07
Bradykinesia (toe tap)	Slope change in frequency	More affected	−0.42	−0.26
Axial	Foot strike angle variability	Less affected	0.41	−0.06
Axial	Lateral step variability	More affected	−0.41	−0.36
Tremor	Rotational rest tremor constancy	Less affected	0.38	−0.03
Axial	Single limb support variability	More affected	0.36	0.10

### 3.3 More affected side measures suffice for both diagnostic and early progression applications in ePD

**Figure 4:**
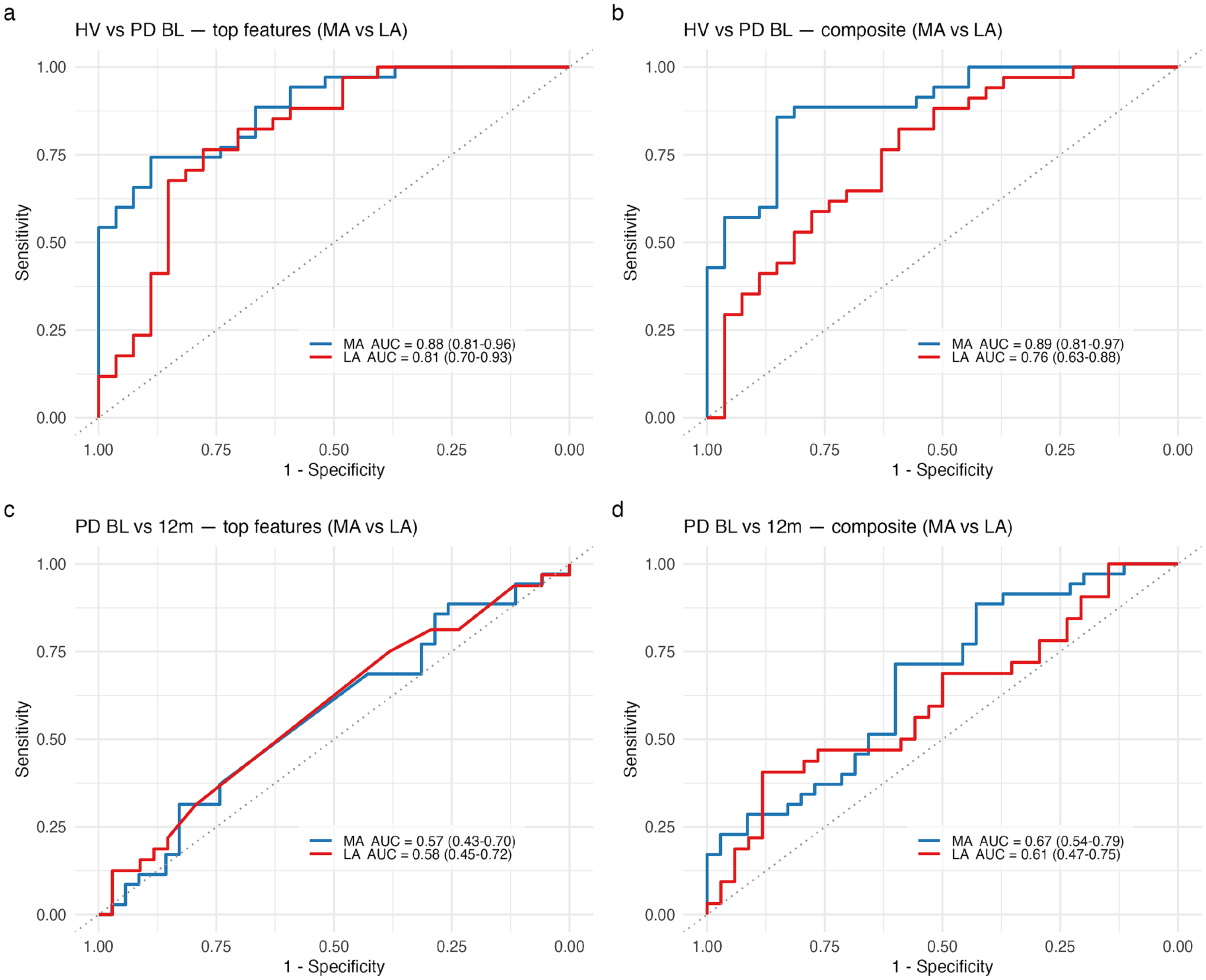
More affected (MA) side measures show superior diagnostic performance; MA and LA side measures perform equivalently for short-term progression detection. **(a,b)** Comparison of MA and LA GLM logistic regression models built using top features or composite score for distinguishing HV and PD at Baseline. **(c,d)** Comparison of MA and LA GLM logistic regression models built using top features or composite score for distinguishing PD at Baseline and 12 month follow-up.

To evaluate the value of MA versus LA side features for diagnosis and progression monitoring, we developed nested-CV models using measures from each side exclusively. For diagnosis, MA-side composite scores showed superior performance (AUC = 0.89, 95% CI 0.81–0.97) compared to LA-side composite scores (AUC = 0.76, 95% CI 0.63–0.88; one-tailed DeLong *p* = 0.040), and MA top-features models trended toward better performance than LA top-features models (MA: AUC = 0.89, 95% CI 0.81–0.96; LA: AUC = 0.81, 95% CI 0.70–0.93; *p* = 0.156) (Figures 4a,b). Notably, combining features from both sides did not improve diagnostic performance over the MA-only models (combined top features AUC = 0.86; combined composite AUC = 0.88; both within MA models’ 95% CIs), as the top features selected by the combined model were predominantly MA-side (Table 1).

For ePD-BL-vs-12 m progression detection, MA and LA side models performed similarly under nested cross-validation, with no statistically significant advantage for either side (top features: MA AUC = 0.57 vs. LA 0.58, one-tailed DeLong *p* = 0.44; composite: MA AUC = 0.67 vs. LA 0.61) (Figures 4c,d). Combined-side models did not improve progression detection beyond the better of the single-side models (combined top features AUC = 0.53, combined composite AUC = 0.56). Overall, MA-side features showed a significant advantage for case–control discrimination at baseline (composite scores p = 0.040), consistent with the asymmetric onset of early-stage PD, and equivalent performance to LA-side features for 12-month progression detection. This pattern suggests that MA-side features alone may be sufficient for both diagnostic and early-progression applications in ePD, simplifying operational logistics for sensor placement and reducing data-collection burden in clinical or trial settings.

## 4 Discussion

Efforts to stage Parkinson’s disease progression increasingly acknowledge the heterogeneity of symptom trajectories and the limitations of relying solely on broad clinical scales (27, 28). To address these challenges, our study investigates granular, domain specific measures objectively quantified with wearable technology, revealing distinct patterns in their diagnostic and progression sensitivity. Our results suggest that using a single model to support both diagnostic and progression endpoints in early PD trials may lead to suboptimal sensitivity. In early Parkinson’s disease (ePD), digital measures most effective for diagnosis were primarily associated with axial and tremor symptoms, whereas measures most sensitive to short-term progression were more often linked to bradykinesia, especially slope-related changes in pronation–supination amplitude, velocity, and frequency over repeated movements. Notably, diagnostic indicators such as arm swing range of motion (Cohen’s *d* = −1.40) and its variability (d = −1.15) showed large baseline differences between ePD and healthy controls but minimal within-subject change over 12 months under paired testing, while progression-linked bradykinesia features detected measurable longitudinal decline (paired d = 0.46–0.67) yet had weaker baseline discriminative power.

Side-specific analysis revealed that more-affected (MA) side measures show a significant advantage over less-affected (LA) side measures for case–control discrimination at baseline (composite scores, one-tailed DeLong *p* = 0.040), reflecting the early emergence of impairment on that side. For 12-month progression detection, MA and LA models performed equivalently under nested cross-validation, suggesting that the asymmetry between sides observed at diagnosis does not translate into a reliable side-specific advantage for short-term longitudinal monitoring in this cohort. From a practical standpoint, this pattern means that focusing on MA-side features alone may be sufficient for both diagnostic and early-progression applications in ePD, simplifying operational logistics for sensor placement and reducing data-collection burden in clinical or trial settings. Together with the domain-focused findings above, these results underscore the importance of side- and domain-aware digital functional measures in revealing nuanced patterns in ePD, while supporting a parsimonious sensor strategy for early-stage trials.

The MDS-UPDRS relies on clinicians to assign ordinal ratings for each item based on multiple observed aspects. For example, item 3.10 (Gait) asks clinicians to integrate stride amplitude, stride speed, foot lift height, heel strike, turning, and arm swing into a single score from 0 (Normal) to 4 (Severe). While this composite approach is valuable for capturing global impairment across the full disease course, it is less sensitive to subtle, short-term changes over one or 2 years. Certain measures, such as arm swing velocity, often change early in the disease and then remain stable, limiting their utility for progression monitoring. Conversely, we observed that heel strike angle variability changes over a one-year interval within ePD, yet it does not differentiate between healthy controls and ePD at baseline. These findings, along with distinct temporal patterns between the more and less affected sides, highlight how standard ordinal ratings can obscure granular progression signals that wearable-sensor derived, side-specific metrics readily capture. While expert neurologists may be adept at recognizing such subtle gait changes and integrating them into clinical ratings, inter-clinician variability in experience can lead to inconsistent sensitivity. Wearable sensors offer an opportunity to provide standardized, objective, and sensitive quantification of these features across sites, reducing variability and enhancing the detection of early progression signals (29–31).

The development of composite biomarkers should be closely aligned to the objectives and stage of disease targeted in a study, recognizing that the measures and weightings most useful for diagnosis may differ from those best suited for progression monitoring (32, 33). The shift in measure relevance over the disease course parallels the model-alignment problem in predictive analytics: highly sensitive models trained to maximize accuracy for one objective (e.g., classification at baseline) may underperform when applied to a different objective (e.g., longitudinal change detection) if their feature sets are not re-selected for the new task. Notably, our nested cross-validation analysis indicated that data-driven feature weighting added little discrimination beyond unweighted z-score summation in this small-cohort setting, suggesting that careful stage-specific feature selection—identifying which measures carry diagnostic vs. progression signal—matters more than the specific scheme used to combine them. In clinical research, failing to address this alignment challenge can result in models that misinterpret stagnant measures as stability or overlook emerging signals specific to a progression phase. Future work investigating adaptive composite designs—where measure selection evolves with disease stage—may mitigate this risk, ensuring that predictive biomarkers remain optimally tuned to the trial’s intent and the biological realities of Parkinson’s progression.

While our findings provide valuable insights into side-dependent and domain focused non-linear changes in digital functional measures, several limitations should be noted. Our dataset consisted of age-matched healthy volunteers and participants with early Parkinson’s disease, followed over a single year. This design limits our ability to capture the full spectrum of symptom trajectories across the complete disease duration or in more diverse patient populations. Throughout the manuscript, we use the term “diagnostic” to denote case–control discrimination between newly diagnosed ePD participants and age-matched healthy volunteers. This does not constitute evaluation of a clinically deployable diagnostic biomarker, since the analysis did not include relevant clinical comparator groups such as essential tremor, atypical Parkinsonism, or non-PD gait disorders; broader differential-diagnosis utility will require dedicated case–control studies against those comparators. Additionally, our measures focused solely on axial, tremor, and bradykinesia domains collected during short, in-clinic assessments. A deeper understanding of progression will require incorporating other symptom domains such as fine motor skills, rigidity, speech (34, 35), and cognition (36), which likely exhibit their own non-linear and stage-dependent changes, as well as alternative methodologies of data collection including passively or via remote assessments. Future studies spanning multiple disease stages, longer follow-up periods, and a broader set of functional measures will be essential to validate and extend these findings.

## Data Availability

The data that support the findings of this study are not openly available due to data privacy controls in place as part of the consortium that this work supports. All data are available to members of the Critical Path for Parkinson’s Consortium 3DT Initiative Stage 2. For those not part of 3DT Stage 2, a proposal may be made to the WATCH-PD Steering Committee (via the corresponding author) for deidentified baseline datasets.
