# Supplementary figures and images for: Stage-specific digital health technology biomarkers enhance diagnostic and early progression detection in Parkinson’s disease
Source: Front Neurol. 2026 Jul 8;17:1869945. doi: 10.3389/fneur.2026.1869945 (PMC13390498; doi:10.3389/fneur.2026.1869945)

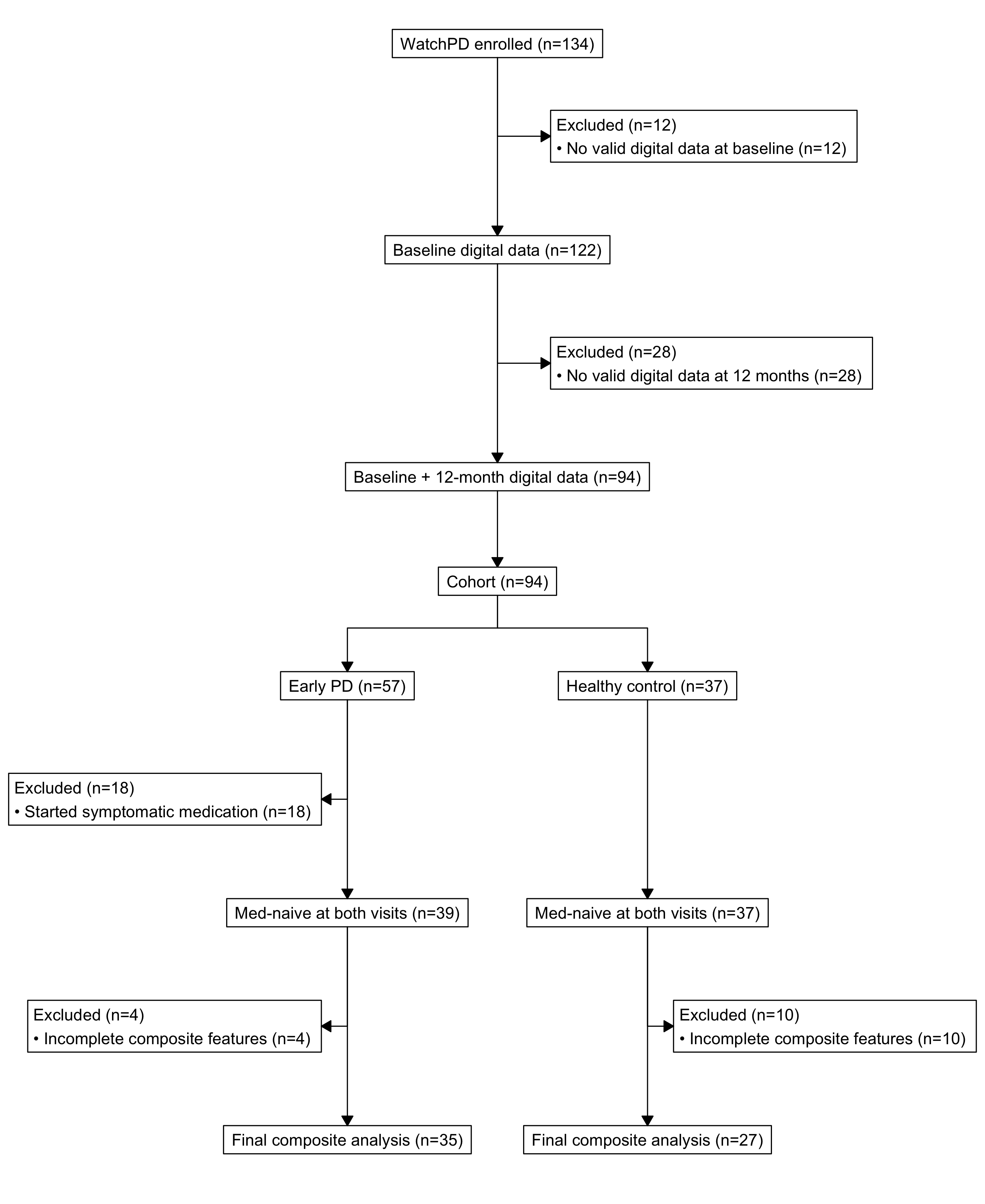

Supplement: Supplementary file 1 [file Image_1.tiff]
